# Structural and functional analysis of tomato sterol C22 desaturase

**DOI:** 10.1186/s12870-021-02898-7

**Published:** 2021-03-17

**Authors:** Laura Gutiérrez-García, Montserrat Arró, Teresa Altabella, Albert Ferrer, Albert Boronat

**Affiliations:** 1 Center for Research in Agricultural Genomics (CSIC-IRTA-UAB-UB), Bellaterra, Barcelona, Spain; 2 Department of Biochemistry and Physiology, Faculty of Pharmacy and Food Sciences, University of Barcelona, 08028 Barcelona, Spain; 3 Department of Biology, Healthcare and the Environment, Faculty of Pharmacy and Food Sciences, University of Barcelona, 08028 Barcelona, Spain; 4 Department of Biochemistry and Molecular Biomedicine, Faculty of Biology, University of Barcelona, 08028 Barcelona, Spain

**Keywords:** Tomato, Sterol metabolism, Stigmasterol, β-Sitosterol, Cytochrome P450, Sterol C22-desaturase, endoplasmic reticulum

## Abstract

**Background:**

Sterols are structural and functional components of eukaryotic cell membranes. Plants produce a complex mixture of sterols, among which β-sitosterol, stigmasterol, campesterol, and cholesterol in some Solanaceae, are the most abundant species. Many reports have shown that the stigmasterol to β-sitosterol ratio changes during plant development and in response to stresses, suggesting that it may play a role in the regulation of these processes. In tomato (*Solanum lycopersicum*), changes in the stigmasterol to β-sitosterol ratio correlate with the induction of the only gene encoding sterol C22-desaturase (C22DES), the enzyme specifically involved in the conversion of β-sitosterol to stigmasterol. However, despite the biological interest of this enzyme, there is still a lack of knowledge about several relevant aspects related to its structure and function.

**Results:**

In this study we report the subcellular localization of tomato C22DES in the endoplasmic reticulum (ER) based on confocal fluorescence microscopy and cell fractionation analyses. Modeling studies have also revealed that C22DES consists of two well-differentiated domains: a single N-terminal transmembrane-helix domain (TMH) anchored in the ER-membrane and a globular (or catalytic) domain that is oriented towards the cytosol. Although TMH is sufficient for the targeting and retention of the enzyme in the ER, the globular domain may also interact and be retained in the ER in the absence of the N-terminal transmembrane domain. The observation that a truncated version of C22DES lacking the TMH is enzymatically inactive revealed that the N-terminal membrane domain is essential for enzyme activity. The in silico analysis of the TMH region of plant C22DES revealed several structural features that could be involved in substrate recognition and binding.

**Conclusions:**

Overall, this study contributes to expand the current knowledge on the structure and function of plant C22DES and to unveil novel aspects related to plant sterol metabolism.

**Supplementary Information:**

The online version contains supplementary material available at 10.1186/s12870-021-02898-7.

## Background

Sterols are isoprenoid-derived lipids that play an essential role in the regulation of membrane fluidity, permeability and function [1, 2]. Sterols share a common structure based on the cyclopentane perhydro phenanthrene ring system with a hydroxyl group at position C3, methyl groups at positions C10 and C13 and a side chain of variable length attached to C17 [3]. Contrary to other eukaryotic organisms, plants are characterized by producing a high diversity of sterols that mostly differ in the nature of the side chain attached to C17, being β-sitosterol, stigmasterol and campesterol the most abundant ones, and in some Solanaceae also cholesterol. In plants, sterols are present in free form and also in conjugated forms as steryl esters, steryl glycosides and acyl steryl glycosides. While free sterols and steryl glycosides are mainly found in the plasma membrane (PM), steryl esters accumulate in cytoplasmic lipid droplets [4].

Sterols are known to be crucial for the function of the PM as they modulate its physicochemical properties, as well as the formation of microdomains (also known as lipid rafts) that are involved in many relevant cellular processes such as cell to cell interactions, signal transduction, membrane transport, protein trafficking and stress responses [5, 6]. In plants, the right function of the PM depends on the balanced levels of campesterol, β-sitosterol and stigmasterol [7]. In particular, changes in the β-sitosterol/stigmasterol ratio have been proposed to influence different developmental processes and stress responses [7]. β-Sitosterol and stigmasterol only differ in the double bond present at position C22 in stigmasterol side chain (Additional file 1: Fig. S1). However, despite their high structural similarity these sterols have a differential effect on the physicochemical properties of the PM [8]. Stigmasterol-enriched membranes are less permeable and, therefore, show a decreased leakage [9, 10]. Therefore, the level of β-stigmasterol in the PM is expected to be tightly regulated during plant development and stress responses [11–13].

Stigmasterol is the end product of the 24-ethyl branch of the sterol biosynthetic pathway and is synthesized from β-sitosterol by the action of the enzyme sterol C22-desaturase (C22DES) (Additional file 1:Fig. S1) [11, 14]. C22DES, also known as CYP710, belongs to the cytochrome P450 (CYP) protein family, which includes enzymes involved in numerous biosynthetic and xenobiotic pathways in all living organisms. CYP proteins share a common catalytic center including a heme-iron binding domain. NADPH acts as the electron donor in the reaction catalyzed by C22DES through the action of cytochrome P450 reductase, a membrane-bound protein localized in the ER membrane [14–16]. C22DES is phylogenetically related to CYP51, a CYP protein having sterol 14-demethylase activity. CYP51 is common to the plant, yeast and animal sterol biosynthetic pathways [17, 18], and there is evolutionary evidence suggesting that these enzymes already existed in the most ancient eukaryotes [19]. Since C22DES acts downstream of CYP51 in the sterol biosynthesis pathway it has been proposed that it evolved from a CYP51 gene duplication [19, 20].

Plant C22DES was cloned and characterized at the biochemical level more than one decade ago [14, 21, 22]. However, there are several functional and structural aspects related to this enzyme that still remain unknown. One of them concerns the elucidation of its subcellular localization, an aspect that is particularly remarkable considering that C22DES may act on β-sitosterol synthesized in the ER during de novo sterol biosynthesis and/or also on β-sitosterol present in the PM (either in free or conjugated form). Although it is widely accepted that free sterol biosynthesis occurs in the ER [23–25], the participation of the PM in the final steps of the sterol pathway has not been excluded [3]. All plant CYP proteins described so far are membrane-bound and mainly localized in the ER. However, some particular CYP proteins have been reported in other subcellular localizations such as mitochondria, plastids and the PM [26, 27]. Therefore, the elucidation of the subcellular localization of C22DES represents a relevant issue in plant sterol metabolism. Other important aspects related to the characterization of C22DES are the identification of structural and functional motifs involved in the intracellular targeting of the enzyme as well as in its membrane topology and catalytic function. Some of these issues have been addressed in the present work using tomato C22DES, which was chosen for this study not only because this plant is one of the most important crops worldwide but also because, in contrast to other plant species, tomato contains a single gene coding for this enzyme [11, 17].

## Results

### Tomato C22DES localizes in the ER

**Fig. 1:**
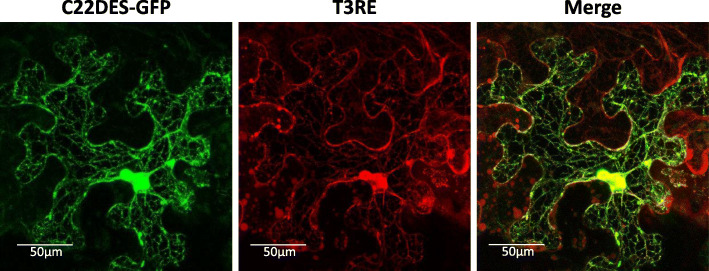
Subcellular localization of C22DES. Confocal optical sections showing the GFP and RFP fluorescence pattern of *N. benthamiana* cells transiently co-expressing the C22DES-GFP fusion protein (left) and the ER marker T3RE (middle). The merge of both images is shown on the right

To define the subcellular location of C22DES, a chimeric protein containing the entire tomato C22DES coding sequence fused at the N-terminal end of the green fluorescent protein (GFP) (C22DES-GFP) was transiently expressed in *Nicotiana benthamiana* leaves. As revealed by confocal fluorescence microscopy, C22DES-GFP exhibited a typical ER-like pattern. This fluorescence pattern was essentially the same observed in cells co-expressing T3RE protein fused to the red fluorescent protein (RFP) (T3RE-RFP), which was used as a specific marker for ER-localization [28] (Fig. 1). Actually, merging the fluorescence of both channels revealed a clear overlap of the two images.

**Fig. 2:**
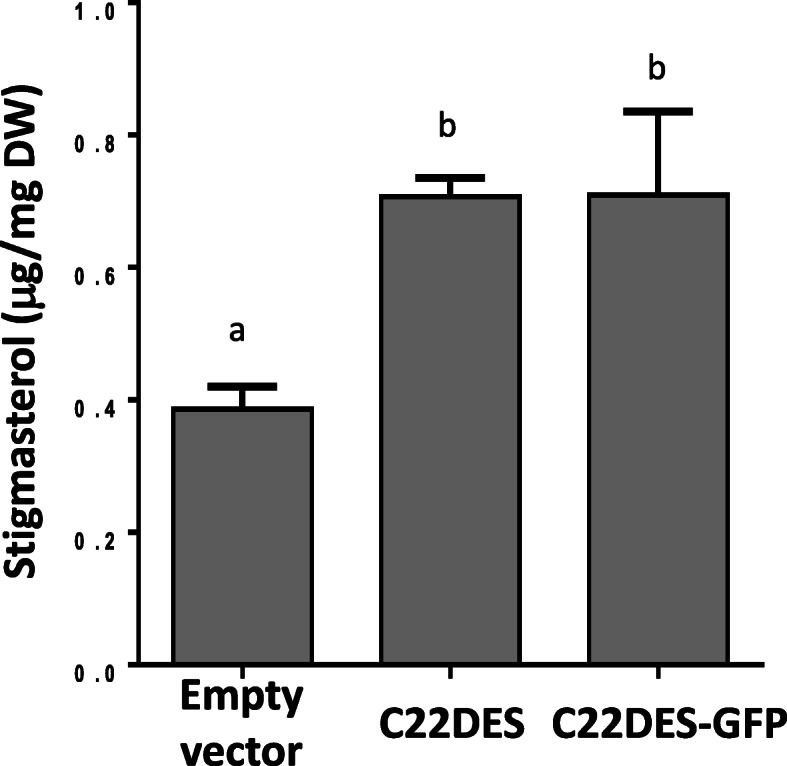
In vivo enzymatic activity of C22DES and C22DES-GFP. Stigmasterol levels in total sterol fractions of *N. benthamiana* leaves transiently expressing C22DES and C22DES-GFP. Values are mean values ± SD of three technical replicates (*n* = 3). Lowercase letters indicate significant differences among mean values relative to those in leaf samples expressing the empty vector (one-way ANOVA with Dunnett’s multiple comparisons test). DW: Dry weight

To make sure that the C-terminal GFP tag was not affecting neither the correct targeting of C22DES nor its catalytic activity, the stigmasterol levels of the agroinfiltrated *N. benthamiana* leaves expressing either the native C22DES or the chimeric C22DES-GFP variant were determined and compared to those of leaves agroinfiltrated with the empty expression vector that were used as a control. As shown in Fig. 2, the total stigmasterol level increased by about 75% in a similar way in both cases. These results revealed that the chimeric enzyme was properly targeted to the subcellular compartment(s) were its substrate (β-sitosterol) is found and also provided an in vivo assay to evaluate the enzyme activity of a set of C22DES-GFP derivatives described below.

### 3D-modeling of tomato C22DES

**Fig. 3:**
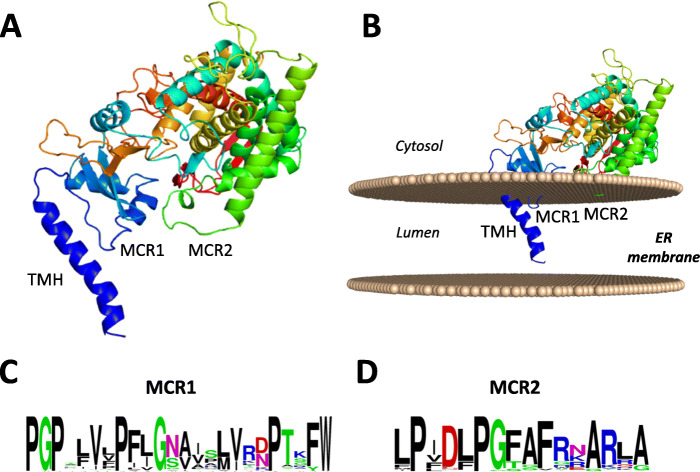
Predicted tomato C22DES tertiary structure. **a** Overall predicted fold of tomato C22DES. **b** Predicted orientation of C22DES in a dioleoylphosphatidylcholine (DOPC) membrane. The predicted N-terminal transmembrane helix (TMH) and the membrane contact regions MCR1 and MCR2 are indicated. **c and d** Sequence logo of the consensus MCR1 and MCR2 sequences obtained from the alignment of the plant C22DES proteins indicated in Additional file 2: Table S1

The tertiary structure of tomato C22DES was modeled with 100% confidence using the single highest scoring template of the *Saccharomyces cerevisiae* lanosterol 14α-demethylase (CYP51) crystal structure [29], which has previously been reported to be an ER-membrane-associated enzyme [30]. The overall tertiary structure predicted for C22DES was very similar to that of yeast CYP51 although a remarkable difference was observed in the N-terminal region. While in CYP51 it is composed by an N-terminal amphipathic α-helix followed by a transmembrane α-helix [29], in C22DES this region is shorter and contains a single putative transmembrane α-helix (residues 1–28), that was referred to as TMH (Fig. 3a). Moreover, an interaction model of C22DES with cell membranes, generated using the PPM web server of the Orientations of Proteins in Membranes (OPM) database [31], predicted two other membrane contact regions in the protein sequence, namely MCR1 (residues 38–58) and MCR2 (residues 227–233) (Fig. 3a and b). MCR1 is a proline-rich motif located in close proximity to TMH and highly conserved among C22DES from different plant species (Fig. 3c) whereas MCR2 is less conserved (Fig. 3d) and localizes in the globular domain next to a long amphipathic α-helix predicted in all plant C22DES (Fig. 3a and b). These results are consistent with our previous finding showing that functional tomato C22DES localizes into the ER membrane of *N. benthamiana* cells.

### TMH is sufficient to target and retain tomato C22DES in the ER membrane

**Fig. 4:**
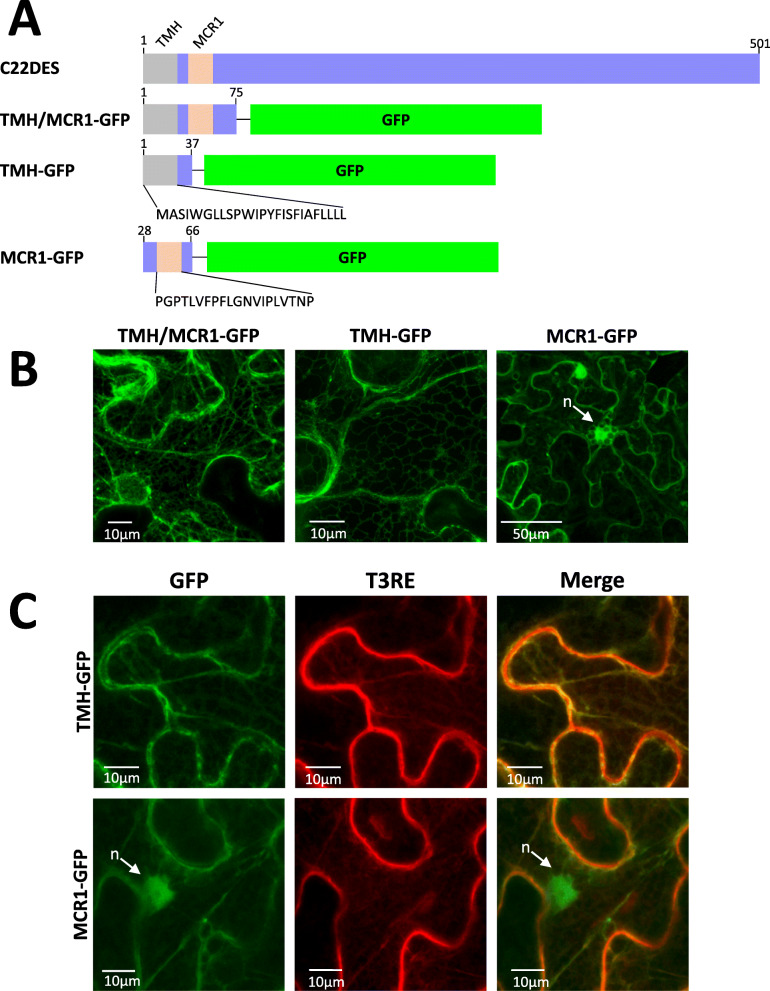
Role of TMH1 in the targeting and retention of C22DES in the ER membrane. **a** Schematic representation of the GFP fusion constructs generated to study the role of TMH and MCR1 in the targeting and retention of C22DES in the ER. Grey boxes indicate the transmembrane helix (TMH), orange boxes correspond to the MCR1 motif and green boxes correspond to the GFP protein. The amino acid sequence of TMH and MCR1 are shown below the corresponding regions. **b** Confocal optical sections showing the GFP fluorescence pattern of *N. benthamiana* cells transiently expressing TMH + MCR1-GFP, TMH-GFP, and MCR1-GFP. The arrow indicates the cell nucleus (n). **c** Close-up view of the fluorescence pattern of TMH-GFP and MCR1-GFP (left), T3RE (middle) and the corresponding merged images (right)

**Fig. 5:**
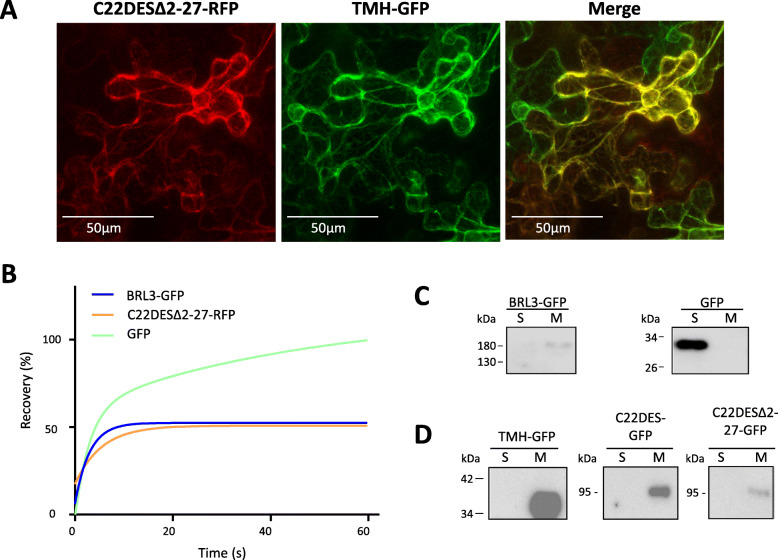
Targeting and retention of the C22DES globular domain in the ER. **a** Confocal optical sections showing the fluorescence of C22DESΔ2–27-RFP (left) and TMH-GFP (middle) transiently expressed in *N. benthamiana* leaves. Merged images are shown on the right. **b** FRAP curves representing the fluorescence recovery rates of C22DESΔ2–27-RFP, BRL3-GFP, and GFP. Fluorescence recovery curves represent the best fits from normalized datasets of at least 6 independently bleached points spots. **(C)** Cropped images of immunoblot analysis of soluble (S) and membrane (M) cell fractions from *N. benthamiana* leaves transiently expressing BRL3-GFP (≈153 kDa) and GFP (≈26.8 kDa) as membrane bound and soluble control proteins, respectively. Full-length blots are presented in Additional file 6: Fig. S4. **c** Cropped images from immunoblot analysis of soluble (S) and membrane (M) cell fractions from *N. benthamiana* leaves transiently expressing C22DESΔ2–27-GFP (≈84.2 kDa), TMH-GFP (≈34 kDa), and C22DES-GFP (≈87.2 kDa). Full-length blots are shown in Additional file 7: Fig. S5

To study the role of the ER interacting sequences predicted in the N-terminal region of tomato C22DES (TMH and MCR1), the amino acid sequences containing residues 1 to 75 (including both TMH and MCR1), residues 1 to 37 (including only TMH), and residues 28 to 66 (including only MCR1) were fused at the N-terminal end of GFP (Fig. 4a). The MCR1 motif was included in these studies because of its close proximity to TMH and its predicted interaction with the ER membrane (Fig. 4b). The subcellular localization of the resulting chimeric proteins (TMH + MCR1-GFP, TMH-GFP, and MCR1-GFP, respectively) was analyzed by confocal microscopy after transient expression in *N. benthamiana* leaves. The fluorescence distribution of TMH + MCR1-GFP and TMH-GFP resulted in a typical ER localization pattern (Fig. 4b). In contrast, MCR1-GFP showed a fluorescence compatible with a cytosolic localization with fluorescence also present the nucleus (Fig. 4b). However, MCR1-GFP also showed some overlap with that of T3RE (Fig. 5c). These results are in agreement with the fact that MCR1 may confer the ability of MCR1-GFP to interact with the ER, reinforcing in this way the functional role of this motif in the interaction of C22DES with the ER. The localization of green fluorescence in the nucleus may be explained considering that at least part of the expressed MCR1-GFP was present in soluble form in the cytosol.

### The globular domain of tomato C22DES interacts with the ER in the absence of TMH

It has been reported that the globular domain of several CYP proteins interacts with the ER membrane in the absence of the transmembrane domain [32–35]. To determine if this is the case in tomato C22DES, a N-terminal truncated form of the enzyme lacking the TMH region (residues 2 to 27) was fused to the N-terminal end of RFP and the resulting protein (C22DESΔ2–27-RFP) transiently expressed along with TMH-GFP in *N. benthamiana* leaves. The fluorescence distribution of C22DESΔ2–27-RFP showed a typical reticular pattern and co-localization with TMH-GFP (Fig. 5a). These results indicated that the globular domain of tomato C22DES was interacting and retained in the ER in the absence of TMH. Fluorescence Recovery After Photobleaching (FRAP) analysis [36, 37] was performed to reinforce these results using the brassinosteroid receptor BRL3 fused to GFP (BRL3-GFP) [38] and GFP as membrane-bound and cytosolic control proteins, respectively. As shown in Fig. 5b, C22DESΔ2–27-RFP showed a recovery rate similar to that of BRL3-GFP, thus confirming its behavior as an integral membrane protein. In agreement with this result, immunoblot analysis using anti-GFP antibodies of the cytosolic and membrane fractions obtained from *N. benthamiana* leaves transiently expressing TMH-GFP, C22DES-GFP and C22DESΔ2–27-GFP demonstrated that they were present in the membrane fraction (Fig. 5c and d). In the immunoblot analysis C22DESΔ2–27-GFP was used instead of C22DESΔ2–27-RFP because anti-GFP antibodies do not recognize the RFP.

### TMH is required for tomato C22DES activity

**Fig. 6:**
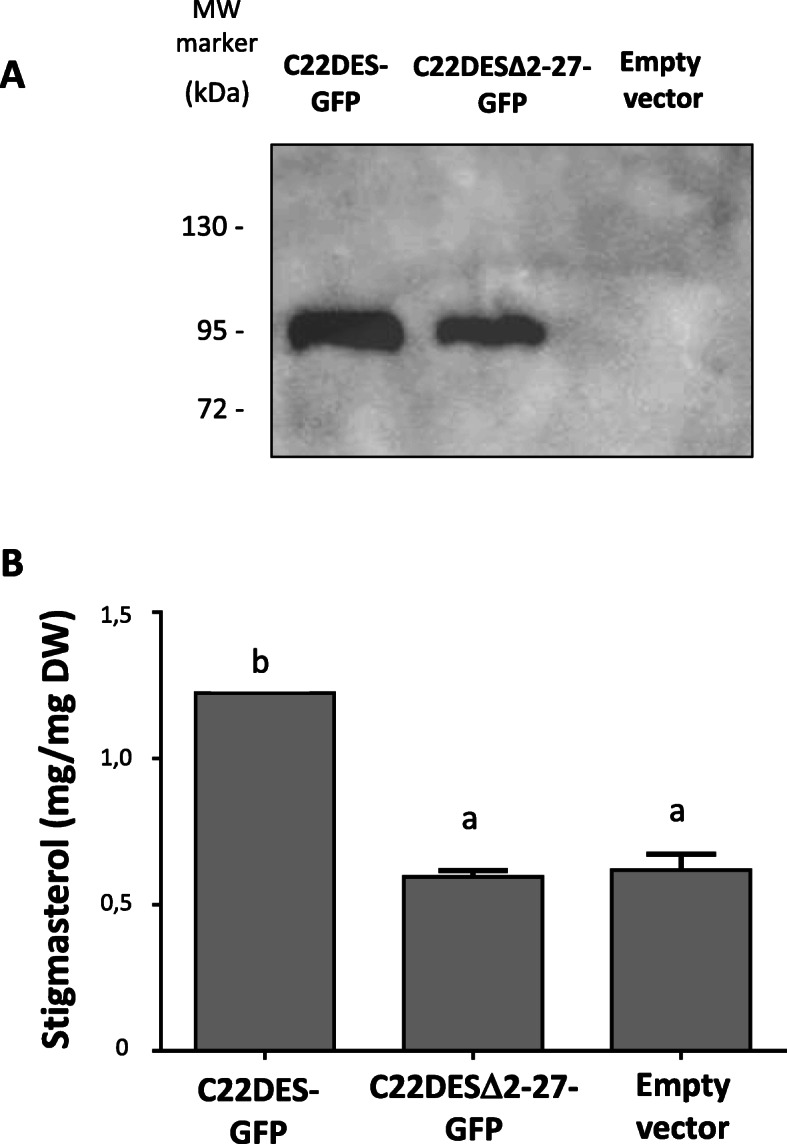
Test of C22DESΔ2–27 enzymatic activity in vivo (**a**) Cropped image from immunoblot analysis of C22DES-GFP (≈87.24 kDa) and C22DESΔ2–27-GFP (≈84.22 kDa) of agroinfiltrated *N. benthamiana* leaves. Full-length blot is presented in Additional file 8: Fig. S6. **b** Stigmasterol levels in total sterol fractions of *N. benthamiana* leaves expressing C22DES-GFP and C22DESΔ2–27-GFP. Values are mean values ± SD of three technical replicates (n = 3). Lowercase letters indicate significant differences among mean values relative to those in leaf samples expressing the empty vector (one-way ANOVA with Dunnett’s multiple comparisons test)

The results reported above suggested that TMH could have other functions in addition to anchor the enzyme to the ER. To explore this possibility, C22DESΔ2–27-GFP was transiently expressed in *N. benthamiana* leaves to evaluate its enzyme activity. C22DES-GFP was expressed in parallel as a positive control. The expression of both proteins was determined by immunoblot analysis using anti-GFP antibodies (Fig. 6a). As expected, the samples expressing C22DES-GFP showed an increase in the total stigmasterol content (Fig. 6b). However, in the case of C22DESΔ2–27-GFP, the stigmasterol content was similar to that found in the leaves agroinfiltrated with the empty expression vector (Fig. 6b), thus indicating that TMH is necessary for enzyme activity.

### The N-terminal region of C22DES from different plant species share features that may be relevant for enzyme activity

**Fig. 7:**
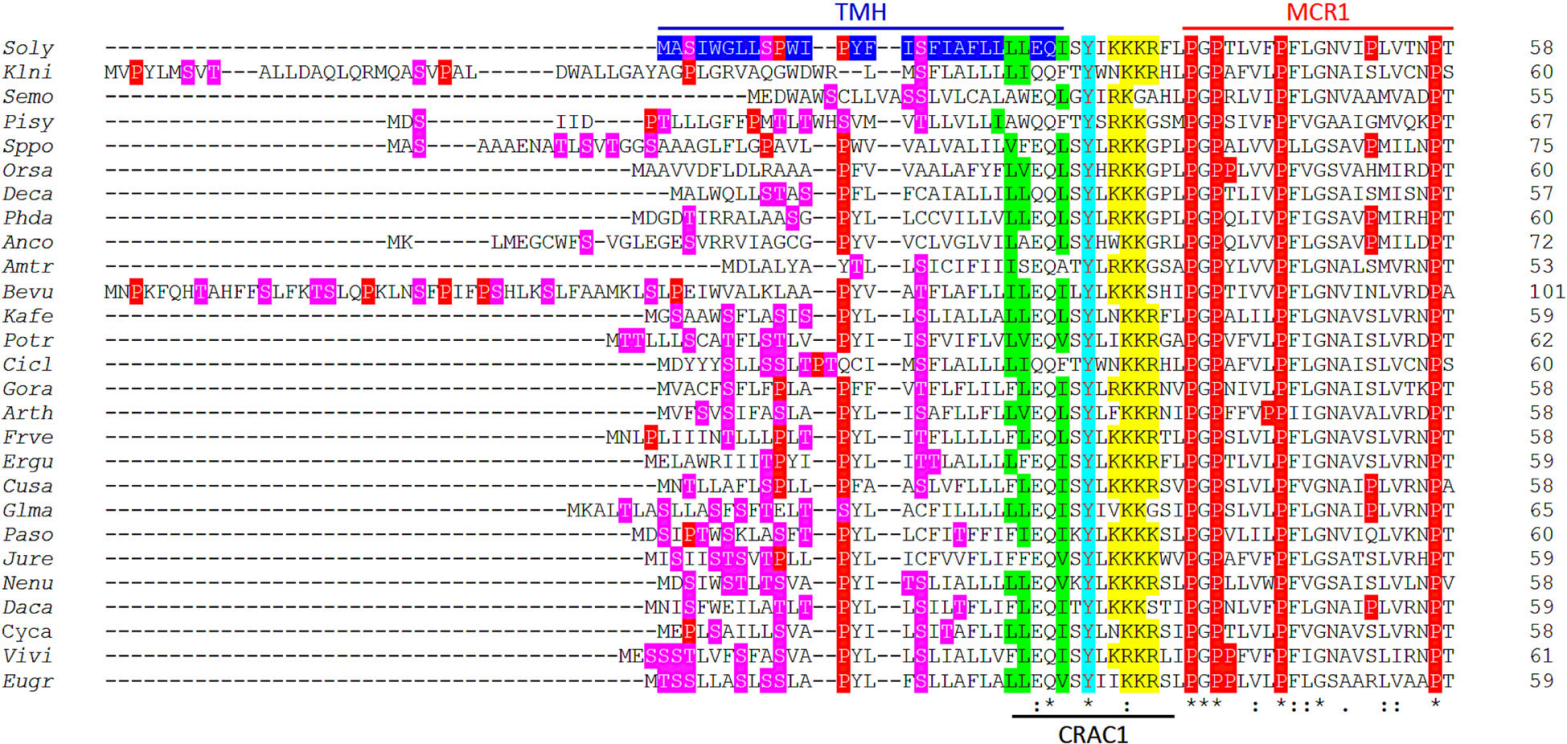
Multiple sequence alignment of the N-terminal region of plant C22DES. The sequence alignment of the N-terminal region of C22DES from the 27 plant species listed in Additional file 2: Table S1 is shown. Amino acid residues are numbered on the right. Asterisks denote residues conserved in all sequences. Colons indicate conservation between amino acid groups of strongly similar properties whereas periods indicate conservation between amino acid groups of weakly similar properties. Hyphens indicate gaps introduced to optimize the alignment. The tomato TMH sequence is highlighted in blue; prolines (P) are shown in red, and serine (S) and threonine (T) residues are shown in magenta. The CRAC1 motif (including the conserved Q27 and Y30 residues) and the MCR1 sequence are also shown. The branched-chain amino-acids [leucine (L), valine (V) and isoleucine (I)] in CRAC1 are shown in green, tyrosine (Y) in cyan and the dibasic residues [arginine (R) and lysine (K)] in yellow. *Soly, Solanum lycopersicum; Klni, Klebsormidium nitens; Semo, Selaginella moellendorffii; Pisy, Pinus sylvestris; Sppo, Spirodela polyrhiza; Orsa, Oryza sativa; Deca, Dendrobium catenatum; Phda, Phoenix dactylifera; Anco, Ananas comosus; Amtr, Amborella trichopoda; Bevu, Beta vulgaris; Kafe, Kalanchoe fedtschenkoi; Potr, Populus trichocarpa; Cicl, Citrus clementina; Gora, Gossypium raimondii; Arth, Arabidopsis thaliana; Frve, Fragaria vesca; Ergu, Erythranthe guttata; Cusa, Cucumis sativus; Glma, Glycine max; Paso, Papaver somniferum; Jure, Juglans regia; Nenu, Nelumbo nucifera; Daca, Daucus carota; Cyca, Cynara cardunculus; Vivi, Vitis vinifera; Eugr, Eucalyptus grandis*

The sequence alignment of C22DES from different plant species showed that both the length and the sequence of the TMH was poorly conserved (Fig. 7). However, a careful inspection of these sequences revealed the presence of several common features shared by all plant C22DES proteins. One of them was the high number of threonine and serine residues in the N-terminal half of THM (Fig. 7). This may be relevant since some studies have described the role of hydroxylated residues in protein transmembrane domains to provide substrate specificity or correct associations with other membrane components through interactions with the hydroxyl group of their polar side chains [39, 40]. Another common feature was the presence of one or more proline residues in the N-terminal half of TMH (Fig. 7). Since proline residues induce a turn of about 30 degrees in α-helices, the TMH sequence of most plant C22DES may have one or more turns in their N-terminal half.

The alignment shown in Fig. 7 also showed the conservation of a glutamine and a tyrosine residue (corresponding to Q27 and Y30 in the tomato sequence) and the presence of a short stretch of positively charged residues between TMH and MCR1. Interestingly, the conserved tyrosine residue and the positively charged residues were identified as elements of a cholesterol recognition/interaction amino acid (CRAC) motif defined by the consensus –L/V-X1–5-Y-X1–5-R/K [41] (Fig. 7). CRAC motifs are usually found in transmembrane helices of membrane proteins. This putative CRAC motif identified in the N-terminal region of C22DES (hereafter referred to as CRAC1) also includes the conserved glutamine residue indicated above.

### The globular domain of plant C22DES contains cholesterol recognition/interaction amino acid consensus motifs

The multiple alignment of plant C22DES (Additional file 2: Table S1) revealed the presence of other conserved cholesterol recognition/interaction amino acid consensus motifs in the globular domain. In addition to the already described CRAC motifs, the globular domain also contains the so called CARC motifs, which correspond to a specular sequence of the CRAC motifs and are defined by the consensus K/R-X1–5-Y/F-X1–5-L/V). CARC motifs may also bind cholesterol although in opposite orientation [42]. Two CRAC motifs (CRAC2 and CRAC4), three CARC motifs (CARC1, CARC2 and CARC3) and one in which a CRAC motif and a CARC motif overlap (CRAC3/CARC4) were found to be conserved in the globular domain of plant C22DES (Additional file 3: Fig. S2). The identification of such a large number of putative cholesterol-interaction motifs was surprising and led to hypothesize that, at least in some cases, it could merely reflect the laxity of these consensus motifs. Since the functional CRAC and CARC motifs reported in the globular domain of membrane proteins are found in α-helices [43, 44] it is likely that only those motifs located in predicted α-helices (CARC2, CARC3, CRAC2 and CRAC3/CARC4) (Additional file 4: Fig. S3) might have a functional role in C22DES. Among them, CARC2, CARC3, and CRAC3/CARC4 could be of special interest as they are present in amphipathic α-helices and located near the catalytic site in the upper part of the globular domain (Additional file 4: Fig. S3).

## Discussion

The results obtained in this work demonstrate that tomato C22DES is an ER-resident protein. The 3D modeling of tomato C22DES using the crystal structure of the phylogenetically related lanosterol 14α-demethylase (CYP51), an enzyme also involved in sterol metabolism thought to be one of the most ancient and conserved P450s across the kingdoms, predicted the presence of an N-terminal hydrophobic transmembrane domain and two short sequences (MCR1 and MCR2) located in the globular domain which also interact with the ER membrane (Fig. 3). Subcellular localization studies using C22DES derivatives fused to GFP indicated that TMH is sufficient for the targeting and retention of C22DES in the ER (Fig. 4 and 5d). These studies also revealed that the globular domain can interact and be retained in the ER membrane in the absence of TMH (Fig. 5). The sequence conservation of MCR1 and MCR2 among plant C22DES (Figs. 3c and d) supports a role for these sequences in the interaction with the ER membrane.

The mechanisms underlying the interaction and retention of the globular domain of C22DES in the ER remain to be characterized. However, it is likely that they could involve the hydrophobic interaction of MCR1 and MCR2 with the ER membrane and/or its interaction with other ER resident proteins such as NADPH-cytochrome P450 reductase, which is required for the function of CYPs [27, 45]. The interaction between some CYP proteins to form heterodimers, as well as between CYPs and other proteins such as cytochrome b5 and UDP-glucuronosyltransferase (UGT)1A has also been reported [46–50].

The observation that the globular domain of tomato C22DES was enzymatically inactive despite behaving as an integral membrane protein (Fig. 6) revealed that TMH was also required for enzyme activity. This result was somehow unexpected considering the recent work of Gnanasekaran et al. (2015) showing that the N-terminal region of CYP720B4, a plant cytochrome P450 involved in isopimeric acid biosynthesis, was not essential for the activity of this enzyme when expressed in *N. benthamiana* leaves. However, the functional role of the N-terminal transmembrane domain of CYPs remains a controversial issue. Thus, while some recombinant CYPs lacking the N-terminal anchor region have been reported to be active in vitro, no activity could be detected in in vivo assays [34, 51, 52]. The differential behavior of the N-terminal region of CYPs may be related, at least in part, with the nature and subcellular availability of their substrates. This would explain why some CYPs show activity under in vitro test conditions in which the substrate is fully available, but not under in vivo assay conditions where the availability of substrate may be a limiting factor. Furthermore, it has been proposed that the N-terminal membrane domain of some CYPs participates in the correct positioning of the globular domain with respect to the membrane during catalysis. Thus, it has been reported that the transient tilting of the globular domain is an essential requirement to allow the interaction of CYPs with their substrates when located within the hydrophobic core of the ER membrane [53, 54]. In the particular case of C22DES, it can be speculated that it could not interact with its substrate (β-sitosterol) unless TMH provides the right anchoring of the globular domain to allow its tilting within the ER membrane during catalysis.

Despite the essential role of TMH in tomato C22DES activity, it was surprising to find that both the length and the amino acid sequence of the N-terminal region of plant C22DES was poorly conserved (Fig. 7). However, a detailed inspection of these sequences revealed two conserved features that could be relevant for enzyme activity: i) the presence of a cholesterol recognition/interaction amino-acid consensus (CRAC) motif, and ii) an enrichment of serine and threonine residues in the N-terminal half of TMH. Cholesterol-binding domains have been the focus of many studies involving computational methods to explore the transmembrane regions of animal proteins for which there is good evidence of their interaction with cholesterol [41, 42]. The first motif to be identified was termed cholesterol recognition/interaction amino-acid consensus (CRAC) and fulfills the consensus (L/V)-X_1–5_-(Y)-X_1–5_-(R/K), (where X is any amino acid) [41, 42, 55]. Another cholesterol-binding motif named CARC corresponds to the mirror version of the CRAC motif with the consensus sequence (K/R)-X_1–5_-(Y/F)-X_1–5_-(L/V) [42, 55]. Despite the CRAC motif was initially identified and characterized in animal proteins [55–58], its cholesterol-binding function has also been demonstrated in plants [59]. Interestingly, a CRAC motif able to interact with β-sitosterol has recently been described in the type 1 cholecystokinin receptor [60]. Thus, it is likely the CRAC1 motif present in the N-terminal region of plant C22DES (CRAC1) may contribute to the interaction of the enzyme with the β-sitosterol present in the ER membrane. Another feature reported in cholesterol-binding regions is the presence of serine and threonine residues. Furthermore, the structural analysis of several cholesterol-binding proteins has led to propose that the hydrophilic side-chain of these amino acids may interact with the C3-hydroxyl group of the cholesterol molecule [61]. Thus, it is likely that the serine and threonine residues present in the N-terminal half of TMH could enhance the interaction of C22DES with β-sitosterol in the ER, facilitating in this way its interaction with the CRAC1 motif.

The localization of C22DES in the ER was not unexpected considering that other enzymes involved in sterol biosynthesis, such as SMT1, CPI1, HYD1, and DWF/DIM [5, 62–64] as well as cytochrome P450 reductase, the physiological redox partner of cytochrome P450s, also localize in this cellular compartment [65]. However, the localization of C22DES in the ER raises the question about how this enzyme can act on the major cellular pool of β-sitosterol present in the PM. It is likely that this process may involve the capacity of the ER to physically interact with the PM at structures known as ER-PM contact sites in which both membranes are in close contact [66–68]. The possibility that enzymes located in one membrane may act on substrates present in a different cell membrane, the so-called *in trans* activity, is not unprecedented [69–72]. Interestingly, specific ER-PM contact sites involved in the regulation of lipid-homeostasis, including phospholipids and sterols, have recently been reported [73]. Furthermore, enzymes involved in lipid biosynthesis have been localized at ER-PM contact sites and shown to contribute to their formation [72, 74]. However, and to the best of our knowledge, the involvement of ER-PM contact sites in sterol or lipid homeostasis has not yet been reported in plants, although it has been suggested that the PM-localized tomato Acyl-CoA:sterol acyltransferase (SlASAT1) may act *in trans* on its substrate cycloartenol in the ER lipid bilayer at ER–PM contact sites to produce cycloartenyl esters [75]. The existence of several conserved CRAC/CARC motifs in the globular domain of plant C22DES suggests that they could have a role in the interaction of the enzyme with the β-sitosterol present in the PM. In this respect, the localization of CARC2, CARC3 and CRAC3/CARC4 in amphypathic α-helices may be relevant considering that this type of structures has been described to serve not only as membrane interaction domains but also as lipid binding sites [76]. The study of the contribution of these CARC and CRAC motifs in the activity of C22DES on the β-sitosterol present in the PM represents an interesting issue in further studies dealing with the functional characterization of this enzyme and the regulation of plant sterol metabolism.

Cellular and metabolic engineering approaches aimed at modifying stigmasterol levels in plants represent a very challenging topic in the field of plant biology and biotechnology [11, 77]. In this sense, the progress in this field should certainly benefit from the new advances in the characterization of C22DES at the genetic, cellular and biochemical levels. Although the structural and functional studies carried out in the present work represent a significant contribution towards this end, we believe that future research in this area should pay special attention to better understand the cellular and molecular mechanisms involved in the regulation of C22DES activity considering the different pools of β-sitosterol present in the plant cell endomembrane system.

## Conclusion

Tomato C22DES is an integral ER-membrane protein having a single transmembrane α-helix at the N-terminal end (TMH). Two short sequences able to interact with the ER membrane (MCR1 and MCR2) have also been predicted in the globular domain. TMH is sufficient for the targeting and retention of the enzyme in the ER-membrane. However, the globular domain can also interact and be retained in the ER-membrane in the absence of TMH, which is required for C22DES activity in vivo. The TMH region contains a highly conserved cholesterol recognition/interaction amino-acid consensus (CRAC) motif and is enriched in threonine and serine residues. These features may be relevant for the recognition and uptake of the β-sitosterol present in the ER membrane to the catalytic site of the enzyme. Overall, the results presented here suggest the existence of a complex pattern of interactions of C22DES with the ER-membrane which are essential for proper enzyme function. The molecular mechanisms underlying the interaction of C22DES with the major cellular pool of β-sitosterol present in the PM remain unknown and deserve further studies.

## Methods

### Plant material

*Nicotiana benthamiana* plants (obtained from the Plant Growth Service of the Center for Research in Agricultural Genomics, Barcelona) were grown under standard greenhouse conditions (14 h light at 26 ± 1 °C and 10 h dark at 21 ± 1 °C) in individual pots of 12 cm of diameter.

### Cloning and plasmid constructions

All the protein-coding sequences lacking the stop codon used for in-frame fusions of tomato C22DES with the GFP and RFP were amplified by PCR using 35S:C22DES plasmid as a template, which was previously obtained in the laboratory and contained the open reading frame coding for the C22DES (GenBank: NM_001247585). All the PCR reactions were performed using high fidelity AccuPrime™ Taq DNA polymerase (Invitrogen) and specific primer pairs (Additional file 5: Table S2). Amplification products were purified and cloned into pDONR207 donor vector using Gateway® technology (Invitrogen) and the resulting pENTRY plasmids transformed into chemically competent Top10 *E. coli* cells, which were used for all cloning steps. The cDNA sequences in the resulting pENTRY plasmids were sequenced to confirm the absence of mutations derived from the amplification process. The verified sequences were sub-cloned into the binary vectors pEarleyGate103 [78] and pGWB454 [79] using Gateway® technology to respectively generate GFP and RFP fusions at the C-terminus under the control of the CaMV35S promoter. The obtained constructs were confirmed by restriction mapping and DNA sequence analysis.

### Agroinfiltration of N. benthamiana leaves

Subcellular localization assays were performed by expression of C22DES fusions with GFP or RFP proteins in leaves of 3–5-week-old *N. benthamiana* plants grown under standard greenhouse conditions (14 h light at 26 ± 1 °C and 10 h dark at 21 ± 1 °C) in individual pots of 12 cm diameter. Leaves were infiltrated with suspensions of the different GV3101 *A. tumefaciens* strains harboring the corresponding recombinant expression plasmids [80, 81], which were prepared as follows. A single positive colony per construct was inoculated into 3 mL of YEB medium supplemented with the right antibiotics (rifampicin 50 μg/ml, gentamycin 25 μg/ml and the plasmid selective antibiotic) and incubated overnight at 28 °C and at 250 rpm in a continuous rotary shaker. A 1:100 dilution of the overnight culture was inoculated into 25 mL of YEB medium containing the same antibiotics and incubated under the same conditions. The culture was centrifuged at 5.000 rpm for 15 min at 4 °C and the bacterial pellets resuspended in infiltration buffer (10 mM MES, pH 5.6, 10 mM MgSO_4_ and 150 μM acetosyringone) to reach a final OD_600_ of 1. Cultures of the transformed *A. tumefaciens* strains were separately mixed with a culture of *A. tumefaciens* strain expressing HC-Pro [82] in a 1:1 ratio and infiltrated in the abaxial part of *N. benthamiana* leaves using a syringe. For co-expression analysis, the strains harboring the different expression plasmids were mixed in equal proportions and also with HC-Pro (the mix never reaching an OD_600_ higher than 1). Agroinfiltrated plants were let to grow for 3–4 days under the greenhouse conditions indicated above.

### Confocal microscopy

Agroinfiltrated leaves were cut into small pieces and the abaxial epidermis analyzed with an Olympus FV 1000 confocal laser-scanning microscope using the 60x water immersion NA: 1.20 objective. The emission windows for fluorescence visualization and the conditions used for image acquisition have been described in Ramírez-Estrada et al. [83]. Fluorescence recovery after photobleaching (FRAP) analysis were also performed as described in Ramírez-Estrada et al. [83]

### Sterol analysis

*N. benthamiana* leaves from three independently agroinfiltrated plants were frozen in liquid nitrogen, ground with a mortar and pestle and lyophilized. Samples (30 mg) were mixed with internal standards [2.5 μg of cholestanol, 5 μg of palmitoyl-cholestanol, 5 μg of cholestanyl-β-D-glucoside and 5 μg of palmitoyl-β-D-glucosyl-cholestanol in chloroform-methanol (2:1)] and extracted with chloroform-methanol (2:1) as indicated in Ramírez-Estrada et al. [83]. Free sterol, steryl ester, steryl glucoside and acyl steryl glcusoside fractions were separated by TLC and their sterol composition determined by GC-MS as described in Lara et al. (2018) [75]. Total stigmaterol levels were calculated from those present in the four sterol fractions extracted from each sample.

### Immunoblot analysis

Soluble (S) and membrane (M) protein fractions from *N. benthamiana* agroinfiltrated leaves were obtained from approximately 10 g of tissue samples as previously reported [83]. Protein concentration was determined as described in Bradford et al.*,* 1976 [84]. Equivalent protein amounts of the T fractions (samples before ultracentrifugation) (around 20 μg), M fractions (around 5 μg) and S fractions (around 20 μg) from each leaf sample were fractionated by 10% polyacrylamide-SDS gel electrophoresis TGX™ FastCast™ Gel (Bio-Rad). After SDS-PAGE, the proteins were transferred to a 0.45 μm nitrocellulose membrane (Amersham, GE Healthcare) using the Trans-Blot® Turbo™ Transfer system (Bio-Rad). Immunoblots using a rabbit anti-GFP antibody (Invitrogen) were performed as described in Ramírez-Estrada et al. [83].

### In silico analysis of protein structure

The 3D structure of the C22DES (NP_001234514.1) was modeled using Phyre2 fold recognition server [85] (http://www.sbg.bio.ic.ac.uk/phyre2). The tertiary structure was predicted using the Lanosterol 14α-Demethylase (Erg11p) of *Saccharomyces cerevisiae* [Protein Data Bank (PDB) ID: c4lxjA] as a template with 100% confidence. For membrane-protein interactions, the predicted 3D models were orientated using PPM web server from the Orientations of Proteins in Membranes (OPM) database [31] (https://opm.phar.umich.edu/ppm_server).

### Protein sequence analysis

*S. lycopersicum* C22DES protein sequence was retrieved from the *SolGenomics Network* website (http://solgenomics.net/) and used as query to search for other plant species homologs using the BLAST tool on the *Phytozome* (https://phytozome.jgi.doe.gov), *GenomeNet* (https://www.genome.jp/), *NCBI* (https://www.ncbi.nlm.nih.gov/), *PLAZA* (https://bioinformatics.psb.ugent.be/plaza/) and the *EnsemblPlants* (http://plants.ensembl.org) websites. The accession numbers of the used homologs are listed in Additional file 2: Table S1. Protein alignments were performed using *ClustalX v 2.0* [86] with default settings and the *GeneDoc* software was used for alignment visualizations and manual edition. The criteria for sequence inclusion on the final alignment was choosing one C22DES from each plant family, preferably those in which *C22DES* was a single-copy gene. For the plant species with more than one C22DES paralog, the selection of just one of them was based on the highest similarity of each paralog with those C22DES from the single-copy species.

For sequence logo generation, WebLogo web server was used [87] (https://weblogo.berkeley.edu/).

## Supplementary Information

**Additional file 1: Supplementary Figure S1**, Reaction catalyzed by C22DES.

**Additional file 2: Supplementary Table S1**, List of plant C22 desaturases used for sequence analysis.

**Additional file 3: Supplementary Figure S2**,Conserved CRAC and CARC motifs present in the globular domain of plant C22DES.

**Additional file 4: Supplementary Figure S3**, Localization of potentially relevant CRAC and CARC motifs in the predicted 3D structure of tomato C22DES.

**Additional file 5: Supplementary Table S2**, Primers and vectors used for preparing the constructs of this study.

**Additional file 6: Supplementary Figure S4**, Full length image of the western blots shown in Fig. 5c.

**Additional file 7: Supplementary Figure S5**, Full length image of the western blots shown in Fig. 5d.

**Additional file 8: Supplementary Figure S6**, Full length image of the western blot shown in Fig. 6.

## Data Availability

All data generated or analysed during this study are included in this published article [and its supplementary information files]. The datasets used and/or analysed during the current study are available from the corresponding author on reasonable request.

## Abbreviations

BLAST: Basic local alignment search tool
C22DES: Sterol C22-desaturase
CARC: CRAC specular sequence
CRAC: Cholesterol recognition/interaction amino acid consensus
CYP: Cytochrome P450
DW: Dry weight
ER: Endoplasmic reticulum
FRAP: Fluorescence recovery after photobleaching
GC-MS: Gas chromatography-mass spectrometry
GFP: Green fluorescent protein
HCPRO: HC-Pro silencing supressor
MCR: Membrane contact region
OD_600_: Optic density at 600 nm
OPM: Orientation of proteins in membranes
PCR: Polymerase chain reaction
PM: Plasma membrane
RFP: Red fluorescent protein
SD: Standard deviation
SDS: Sodium Dodecyl Sulfate
SDS-PAGE: SDS-Polyacrylamide Gel Electrophoresis
TLC: Thin layer chromatography
TMH: Transmembrane helix motif
