# Supplementary material for: Structural and functional analysis of tomato sterol C22 desaturase
Source: BMC Plant Biol. 2021 Mar 17;21:141. doi: 10.1186/s12870-021-02898-7 (PMC7972189; doi:10.1186/s12870-021-02898-7)
Supplement: Supplementary file 1 — Additional file 1: Supplementary Figure S1, Reaction catalyzed by C22DES. [file 12870_2021_2898_MOESM1_ESM.pdf]

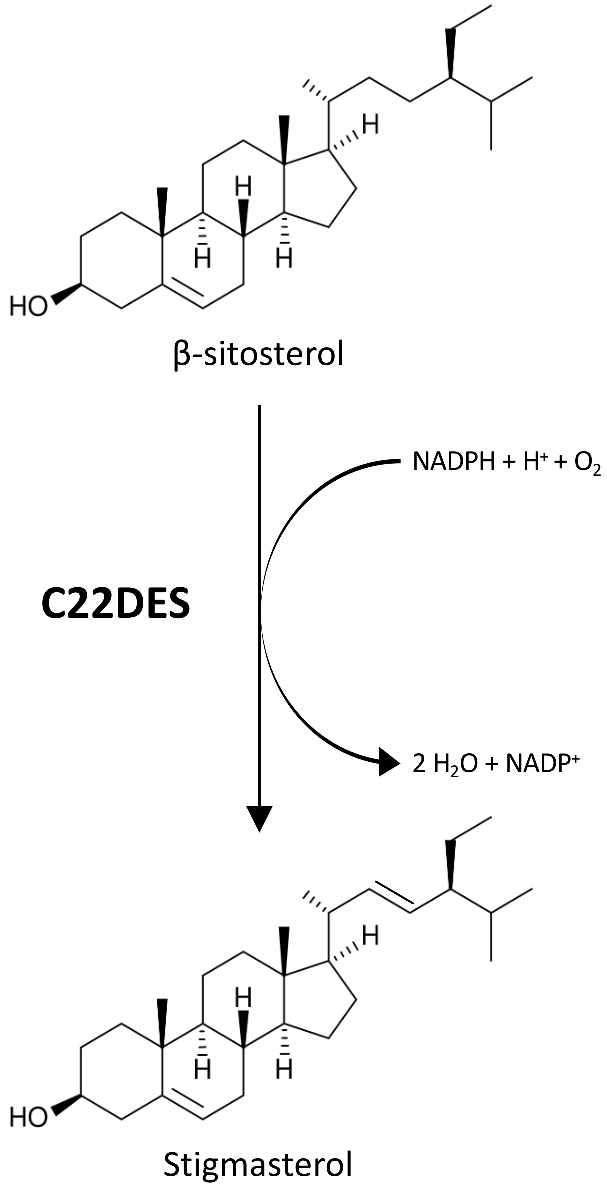

Figure S1. Reaction catalyzed by C22DES**.** Like in other CYP catalyzed reactions NADPH acts as the electron donor through the action of cytochrome P450 reductase (not shown).
