# Supplementary material for: Structural and functional analysis of tomato sterol C22 desaturase
Source: BMC Plant Biol. 2021 Mar 17;21:141. doi: 10.1186/s12870-021-02898-7 (PMC7972189; doi:10.1186/s12870-021-02898-7)
Supplement: Supplementary file 2 — Additional file 2: Supplementary Table S1, List of plant C22 desaturases used for sequence analysis. [file 12870_2021_2898_MOESM2_ESM.pdf]

Table S1. List of plant C22 desaturases used for sequence analysis

Species	Family	Gene ID	Gene Source	Uniprot code
Amaranthus hypochondriacus	Amaranthaceae	AHYPO_007104-RA	Phytozome	-
Beta vulgaris	Amaranthaceae	104883528	NCBI	A0A0J8B8L5_BETVU
Spinacia oleracea	Amaranthaceae	110778797	NCBI	-
Amborella trichopoda	Amborellaceae	18435199	NCBI	W1PIR2_AMBTC
Daucus carota	Apiaceae	108199974	NCBI	A0A175YLR6_DAUCA
Elaeis guineensis	Arecaceae	105048162	NCBI	-
Phoenix dactylifera	Arecaceae	103703021	NCBI	A0A2H3XI04_PHODC
Asparagus officinalis	Asparagaceae	109828814	NCBI	-
Cynara cardunculus	Asteraceae	112504656	NCBI	A0A103RTL5_CYNCS
Helianthus annuus	Asteraceae	110937398	NCBI	A0A251V0G5_HELAN
Lactuca sativa	Asteraceae	111890946	NCBI	A0A2J6MAQ4_LACSA
Arabidopsis lyrata	Brassicaceae	9317406	NCBI	D7LH13_ARALL
Arabidopsis thaliana	Brassicaceae	818013	NCBI	C7101_ARATH
Boechera stricta	Brassicaceae	Bostr.23794s0661	Phytozome	-
Brassica oleracea	Brassicaceae	106339917	NCBI	A0A0D3BS57_BRAOL
Brassica rapa	Brassicaceae	103867449	NCBI	A0A397Z8B6_BRACM
Capsella grandiflora	Brassicaceae	Cagra.7352s0002	Phytozome	-
Capsella rubella	Brassicaceae	17887986	NCBI	R0FZJ8_9BRAS
Eutrema salsugineum	Brassicaceae	18026553	NCBI	V4MHN1_EUTSA
Ananas comosus	Bromeliaceae	109716120	NCBI	A0A199UT02_ANACO
Carica papaya	Caricaceae	110807637	NCBI	-
Chlamydomonas reinhardtii	Chlamydomonaceae	5726941	NCBI	A8JF53_CHLRE
Tarenaya hassleriana	Cleomaceae	104814293	NCBI	-
Ipomoea nil	Convolvulaceae	109192033	NCBI	-
Kalanchoe fedtschenkoi	Crassulaceae	Kaladp0840s0006	Phytozome	-
Kalanchoe laxiflora	Crassulaceae	Kalax.0554s0026	Phytozome	-
Cucumis sativus	Cucurbitaceae	101221194	NCBI	A0A0A0KKM3_CUCSA
Dunaliella salina	Dunaliellaceae	Dusal.0187s00006.1	Phytozome	-
Manihot esculenta	Euphorbiaceae	110621253	NCBI	A0A2C9WLR2_MANES
Glycine max	Fabaceae	100808148	NCBI	I1MF42_SOYBN
Medicago truncatula	Fabaceae	11437817	NCBI	Q2MIZ9_MEDTR
Phaseolus vulgaris	Fabaceae	18629002	NCBI	V7BPJ1_PHAVU
Trifolium pratense	Fabaceae	Tp57577_TGAC_v2_mRNA36715	Phytozome	-
Quercus suber	Fagaceae	112009839	NCBI	-
Physcomitrella patens	Funariaceae	112285903	NCBI	A0A2K1L7U2_PHYPA
Juglans regia	Juglandaceae	109021374	NCBI	A0A2I4HTQ7_JUGRE
Klebsormidium nitens	Klebsormidiaceae	-	-	A0A1Y1IJF8_KLENI
Spirodella polyrhiza	Lemnaceae	Spipo8G0071000	Phytozome	-
Linum usitatissimum	Linaceae	Lus10028129.g	Phytozome	-
Gossypium raimondii	Malvaceae	105769297	NCBI	A0A0D2QGW2_GOSRA
Theobroma cacao	Malvaceae	18596516	NCBI	A0A061GD05_THECC
Marchantia polymorpha	Marchantiaceae	Mapoly0103s0038	Phytozome	A0A2R6WDZ6_MARPO
Eucalyptus grandis	Myrtaceae	104440796	NCBI	A0A059CC63_EUCGR
Nelumbo nucifera	Nelumbonaceae	104597733	NCBI	A0A1U7ZZG5_NELNU
Olea europaea	Oleaceae	111407278	NCBI	-

Dendrobium catenatum	Orchidaceae	110110071	NCBI	A0A2I0X332_9ASPA
Papaver somniferum	Papaveraceae	113345304	NCBI	-
Sesamum indicum	Pedaliaceae	105172499	NCBI	-
Erythranthe guttata	Phrymaceae	105951371	NCBI	A0A022RRY1_ERYGU
Pinus pinaster	Pinaceae	PSY00016483	PLAZA	-
Pinus sylvestris	Pinaceae	PPI00012102	PLAZA	-
Brachypodium distachyon	Poaceae	100827562	NCBI	-
Brachypodium stacei	Poaceae	Brast01G342000	Phytozome	-
Oryza sativa	Poaceae	4325564	NCBI	Q0JPP0_ORYSJ
Panicum hallii	Poaceae	112893543	NCBI	A0A2T7DST8_9POAL
Setaria italica	Poaceae	101764868	NCBI	K4A8R3_SETIT
Setaria viridis	Poaceae	Sevir.9G372000	Phytozome	-
Sorghum bicolor	Poaceae	8079522	NCBI	C5XL37_SORBI
Zea mays	Poaceae	107305676	NCBI	A0A1D6MG27_MAIZE
Aquilegia caerulea	Ranunculaceae	AQUCO_02800015v1	Phytozome	A0A2G5D3K5_AQUCA
Fragaria vesca	Rosaceae	105350241	NCBI	-
Malus domestica	Rosaceae	103434989	NCBI	-
Citrus clementina	Rutaceae	18050706	NCBI	V4TQW1_9ROSI
Citrus sinensis	Rutaceae	102618952	NCBI	A0A067GRV9_CITSI
Populus trichocarpa	Salicaceae	7472572	NCBI	B9H3Z9_POPTR
Salix purpurea	Salicaceae	SapurV1A.0560s0090	Phytozome	-
Selaginella moellendorffii	Selaginellaceae	9633545	NCBI	D8QPW3_SELML
Solanum lycopersicum	Solanaceae	100136886	NCBI	A9QPL5_SOLLC
Solanum tuberosum	Solanaceae	102581211	NCBI	M1BWG7_SOLTU
Sphagnum fallax	Sphagnaceae	Sphfalx0121s0046	Phytozome	-
Vitis vinifera	Vitaceae	100255505	NCBI	F6HLR0_VITVI
Volvox carteri	Volvocaceae	9623124	NCBI	D8UHA1_VOLCA
Zostera marina	Zosteraceae	ZOSMA_88G00340	Phytozome	A0A0K9NKC2_ZOSMR
