# Supplementary material for: Structural and functional analysis of tomato sterol C22 desaturase
Source: BMC Plant Biol. 2021 Mar 17;21:141. doi: 10.1186/s12870-021-02898-7 (PMC7972189; doi:10.1186/s12870-021-02898-7)
Supplement: Supplementary file 3 — Additional file 3: Supplementary Figure S2,Conserved CRAC and CARC motifs present in the globular domain of plant C22DES. [file 12870_2021_2898_MOESM3_ESM.pdf]

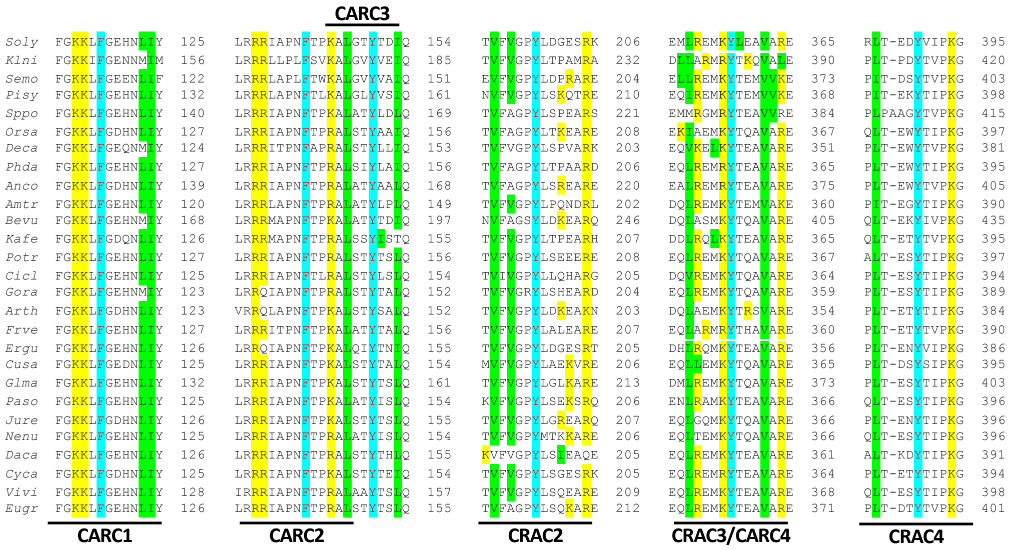

Figure S2. Conserved CRAC and CARC motifs present in the globular domain of plant C22DES. Amino acid residues are numbered on the right side of each motif. Branched-chain amino-acids [leucine (L), valine (V) and isoleucine (I)] are shown in green, tyrosine (Y) in cyan and the dibasic residues [arginine (R) and lysine (K)] in yellow.
