# Supplementary material for: Structural and functional analysis of tomato sterol C22 desaturase
Source: BMC Plant Biol. 2021 Mar 17;21:141. doi: 10.1186/s12870-021-02898-7 (PMC7972189; doi:10.1186/s12870-021-02898-7)
Supplement: Supplementary file 4 — Additional file 4: Supplementary Figure S3, Localization of potentially relevant CRAC and CARC motifs in the predicted 3D structure of tomato C22DES. [file 12870_2021_2898_MOESM4_ESM.pdf]

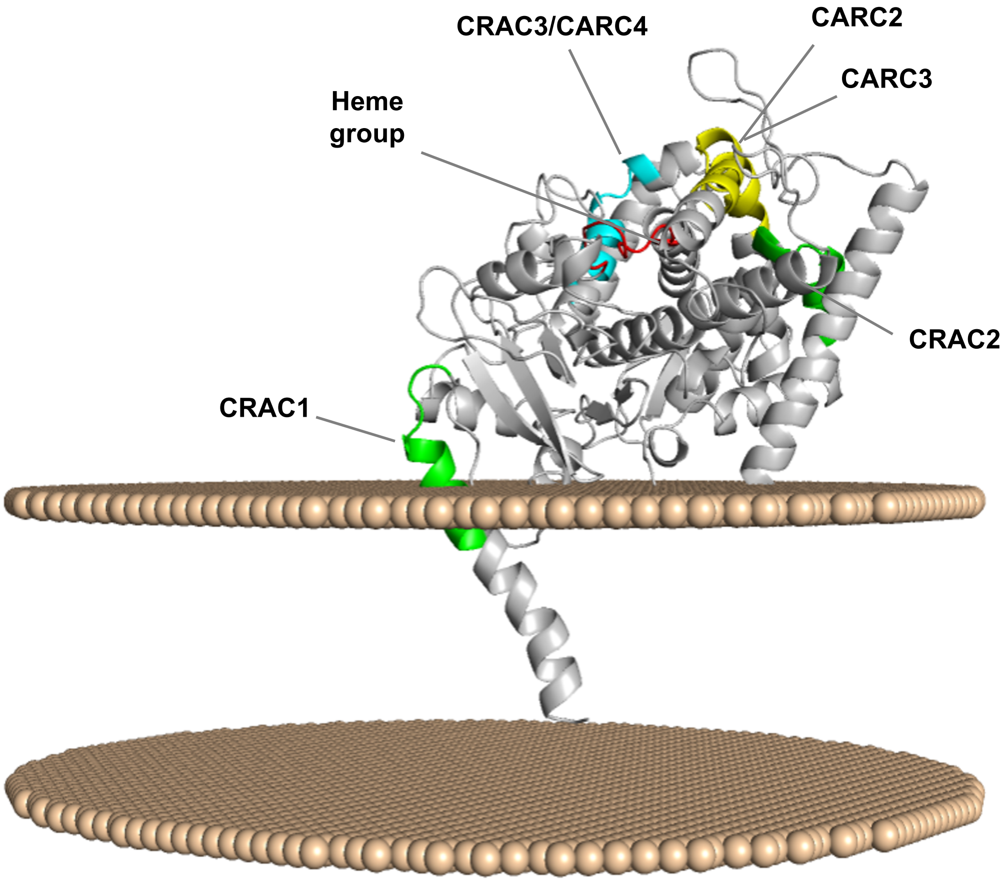

Figure S3. Localization of potentially relevant CRAC and CARC motifs in the predicted 3D structure of tomato C22DES. The position of the heme group in the catalytic site is also shown.
