# Supplementary material for: Structural and functional analysis of tomato sterol C22 desaturase
Source: BMC Plant Biol. 2021 Mar 17;21:141. doi: 10.1186/s12870-021-02898-7 (PMC7972189; doi:10.1186/s12870-021-02898-7)
Supplement: Supplementary file 5 — Additional file 5: Supplementary Table S2, Primers and vectors used for preparing the constructs of this study. [file 12870_2021_2898_MOESM5_ESM.pdf]

Table S2. Primers and vectors used for preparing the constructs of this study. ATTB recombination sites are shown in bold, and the ATG start codons are underlined.

Construct	Template	Primers	Vector
35S:C22DES	cDNA	-	pKGW
C22DES-GFP	35S:C22des	**GGGGACAAGTTTGTACAAAAAAGCAGGCT**TCATGGCATCCATTTGGGGTTTGTTATC<br>**GGGGACCACTTTGTACAAGAAAGCTGGGT**CTCGTGTGCACCTGTGTGCAAG	pEarleyGate103
TMH-GFP	35S:C22des	**GGGGACAAGTTTGTACAAAAAAGCAGGCT**TCATGGCATCCATTTGGGGTTTGTTATC<br>**GGGGACCACTTTGTACAAGAAAGCTGGGT**CAAGAAAACGCTTCTTCTTGATGTAAGAGAT	pEarleyGate103
MCR1-GFP	35S:C22des	**GGGGACAAGTTTGTACAAAAAAGCAGGCT**TCATGATCTCTTACATCAAGAAGAAGCGT<br>**GGGGACCACTTTGTACAAGAAAGCTGGGT**CTGATTGAAGGTCCCAGAATTTAGTTGG	pEarleyGate103
TMH+MCR1-GFP	35S:C22des	**GGGGACAAGTTTGTACAAAAAAGCAGGCT**TCATGGCATCCATTTGGGGTTTGTTATC<br>**GGGGACCACTTTGTACAAGAAAGCTGGGT**CTGATTGAAGGTCCCAGAATTTAGTTGG	pEarleyGate103
C22DESΔ2-27-RFP	35S:C22des	**GGGGACCACTTTGTACAAGAAAGCTGGGT**CTCGTGTGCACCTGTGTGCAAG<br>**GGGGACAAGTTTGTACAAAAAAGCAGGCT**TCATGATCTCTTACATCAAGAAGAAGCGTTTTCTTC	pGWB454
C22DESΔ2-27-GFP	35S:C22des	**GGGGACCACTTTGTACAAGAAAGCTGGGT**CTCGTGTGCACCTGTGTGCAAG<br>**GGGGACAAGTTTGTACAAAAAAGCAGGCT**TCATGATCTCTTACATCAAGAAGAAGCGTTTTCTTC	pEarleyGate103
