# Supplementary material for: Structural and functional analysis of tomato sterol C22 desaturase
Source: BMC Plant Biol. 2021 Mar 17;21:141. doi: 10.1186/s12870-021-02898-7 (PMC7972189; doi:10.1186/s12870-021-02898-7)
Supplement: Supplementary file 6 — Additional file 6: Supplementary Figure S4, Full length image of the western blots shown in Fig. 5c. [file 12870_2021_2898_MOESM6_ESM.pdf]

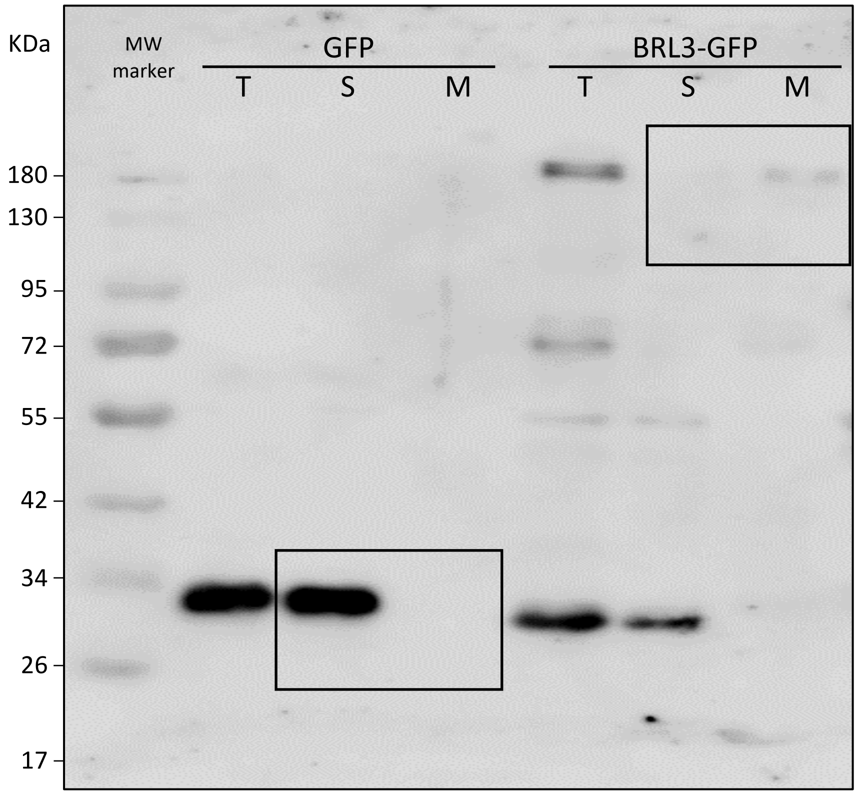

Figure S4. Full length image of the western blots shown in Fig. 5C. Immunoblot analysis of total (T), soluble (S), and membrane (M) cell fractions from *N. benthamiana* leaves expressing BRL3-GFP (≈153 KDa) and GFP (≈26.85 KDa) used as membrane-bound and soluble control proteins, respectively.
