# Supplementary material for: Structural and functional analysis of tomato sterol C22 desaturase
Source: BMC Plant Biol. 2021 Mar 17;21:141. doi: 10.1186/s12870-021-02898-7 (PMC7972189; doi:10.1186/s12870-021-02898-7)
Supplement: Supplementary file 7 — Additional file 7: Supplementary Figure S5, Full length image of the western blots shown in Fig. 5d. [file 12870_2021_2898_MOESM7_ESM.pdf]

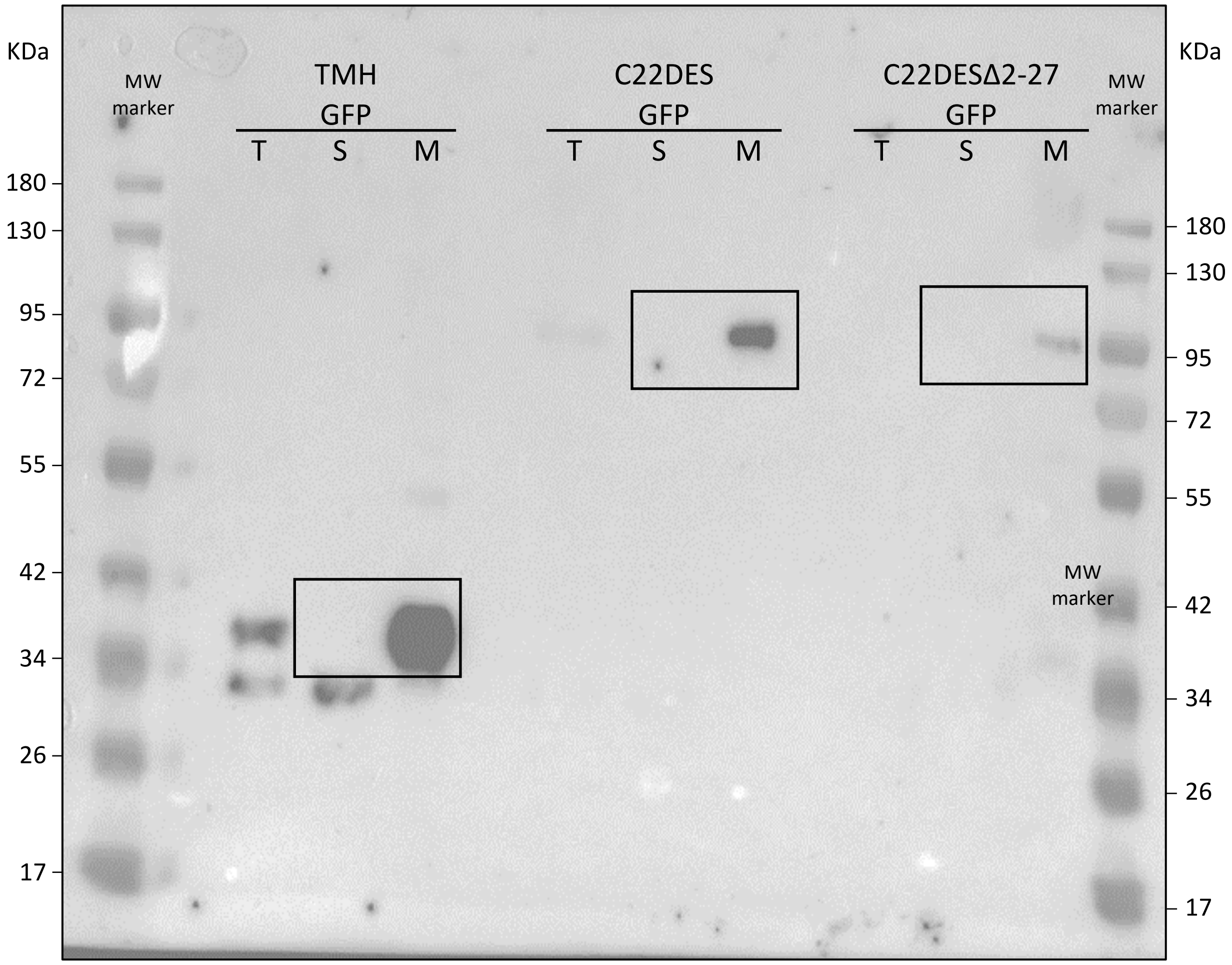

Figure S5. Full length image of the western blots shown in Fig. 5D. Immunoblot analysis of total (T), soluble (S) and membrane (M) cell fractions from *N. benthamiana* leaves expressing C22DESΔ2-27-GFP (≈84.22 kDa), TMH-GFP (≈34.06 kDa), and C22DES-GFP (≈87.24 kDa).
