# Supplementary material for: Structural and functional analysis of tomato sterol C22 desaturase
Source: BMC Plant Biol. 2021 Mar 17;21:141. doi: 10.1186/s12870-021-02898-7 (PMC7972189; doi:10.1186/s12870-021-02898-7)
Supplement: Supplementary file 8 — Additional file 8: Supplementary Figure S6, Full length image of the western blot shown in Fig. 6. [file 12870_2021_2898_MOESM8_ESM.pdf]

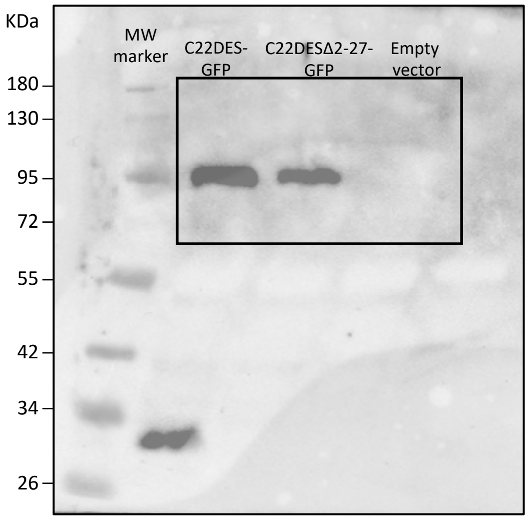

Figure S6. Full length image of the western blot shown in Fig. 6. Immunoblot analysis of C22DES-GFP (≈87.2 KDa) and C22DESΔ2-27-GFP (≈84.2 KDa) levels in agroinfiltrated *N. benthamiana* leaves
